# The effects of a caffeine-like supplement, TeaCrine®, on muscular strength, endurance and power performance in resistance-trained men

**DOI:** 10.1186/s12970-019-0316-5

**Published:** 2019-10-28

**Authors:** Kyle R. Cesareo, Justin R. Mason, Patrick G. Saracino, Margaret C. Morrissey, Michael J. Ormsbee

**Affiliations:** 1 Department of Nutrition, Food & Exercise Sciences, Institute of Sports Sciences & Medicine, Florida State University, 1104 Spirit Way, Tallahassee, FL 32306 USA; 2 The Center for Applied Health Sciences, Canfield, OH 44515 USA; 3 Deparment of Occupational Therapy, University of Florida, Gainesville, FL 32611 USA; 4 Korey Stringer Institute, Department of Kinesiology, University of Connecticut, Storrs, CT 60268 USA; 5 Discipline of Biokinetics, Exercise and Leisure Sciences, University of KwaZulu-Natal, Durban, South Africa

**Keywords:** Caffeine, TeaCrine®, Bench press, Squat, Ergogenic, Strength, Power, Endurance, Supplements

## Abstract

**Background:**

TeaCrine® is the synthetic version to naturally occurring theacrine (1, 3, 7, 9-tetramethyluric acid) found in the leaves of *Camellia* kucha tea plants. A few studies have examined the effects of TeaCrine® on cognitive perception, but no research exists examining its effects on resistance exercise performance. The purpose of this study was to determine the efficacy of TeaCrine®, a caffeine-like compound, on maximal muscular strength, endurance, and power performance in resistance-trained men.

**Methods:**

Twelve resistance-trained men participated in a randomized, double-blind, cross-over designed study. Each participant performed one-repetition maximum (1RM) bench press, 1RM squat, bench press repetitions to failure (RTF) at 70% 1RM, squat RTF at 70% 1RM, and 2-km rowing time trial 90 min after consumption of: (1) Caffeine 300 mg (CAFF300); (2) TeaCrine® 300 mg (TEA300); (3) TeaCrine® + Caffeine (COMBO; 150 mg/150 mg); (4) Placebo 300 mg (PLA). Power and velocity were measured using a TENDO Power Analyzer. Visual analogue scales for energy, focus, motivation to exercise, and fatigue were administered at baseline and 90 min post-treatment ingestion (pre-workout). Rating of perceived exertion was assessed after bench press RTF and squat RTF.

**Results:**

There were no differences between groups for 1RM, RTF, and power in the bench press and squat exercises. Only CAFF300 resulted in significant increases in perceived energy and motivation to exercise vs. TEA300 and PLA (Energy: + 9.8%, 95% confidence interval [3.3–16.4%], *p* < 0.01; + 15.3%, 95% CI [2.2–28.5%], *p* < 0.02; Motivation to exercise: + 8.9%, 95% CI [0.2–17.6%], *p* = 0.04, + 14.8%, 95% CI [4.7–24.8%], *p* < 0.01, respectively) and increased focus (+ 9.6%, 95% CI [2.1–17.1%], *p* = 0.01) vs. TEA300, but there were no significant differences between CAFF300 and COMBO (Energy + 3.9% [− 6.9–14.7%], Focus + 2.5% [− 6.3–11.3%], Motivation to exercise + 0.5% [− 11.6–12.6%]; *p* > 0.05).

**Conclusion:**

Neither TEA300, CAFF300, COMBO, or PLA (when consumed 90 min pre-exercise) improved muscular strength, power, or endurance performance in resistance-trained men. Only CAFF300 improved measures of focus, energy, and motivation to exercise.

## Background

The ergogenic properties of caffeine have spurred the production of caffeine products and caffeine-like compounds. One such caffeine-like compound is TeaCrine®, the nature identical, bio-active version of theacrine (1, 3, 7, 9-tetramethyluric acid), which is believed to act in a similar manner to caffeine, as an adenosine receptor antagonist [1]. Utilizing a rat model, Feduccia et al. [1] reported an increase in locomotor activity following theacrine administration, similar to other neuroactive agents such as caffeine, but unlike caffeine, repeated doses of theacrine did not result in a habituation effect to locomotor activity. Additionally, theacrine has been reported to positively alter mood and fatigue and exhibit anti-inflammatory and analgesic properties in rats [2, 3]. Limited studies exist which examine TeaCrine® supplementation in humans [4–8], but it appears to positively influence cognitive perceptions of energy, focus, and motivation, similarly to caffeine, while notably unaltering hemodynamics (blood pressure, heart rate) which has been reported with caffeine use [9]. The unaltering hemodynamics with TeaCrine® use poses an interesting concept: if TeaCrine® can exert similar or enhanced ergogenic effects to that of caffeine without the possible side effects reported with caffeine use, then TeaCrine® may be an applicable replacement supplement for caffeine, or, a lower dose of caffeine can be used in conjunction with TeaCrine® to mitigate/minimize unwanted side effects while utilizing elicited ergogenic effects. While appearing favorable to influence cognitive perceptions, no human data exists observing the effects of TeaCrine® on resistance exercise performance. Caffeine (1, 3, 7-trimethylxanthine) is a methylxanthine that exerts a stimulatory effect through stimulation of the central nervous system (CNS). A plethora of research exists reporting both ergogenic effects and no effect of caffeine on muscular endurance [10–21], strength [12–15, 20–26], and power [15, 27–30]. Given the similarities between TeaCrine® and caffeine, and the absence of resistance exercise data with TeaCrine®, the purpose of this study was to examine the effects of 300 mg TeaCrine® (TEA300), 150 mg Caffeine + 150 mg TeaCrine® (COMBO), and 300 mg Caffeine (CAFF300) compared to placebo (PLA) on muscular strength (1RM), endurance (repetitions to failure [RTF] at 70% 1RM), and power in the bench press and squat exercises, wherein a 300 mg dose of caffeine was used to match the 150/150 mg Caffeine/TeaCrine® blend. Further, we sought to examine the effects of these supplements on subjective measures of energy, focus, motivation to exercise, fatigue, and rating of perceived exertion (RPE). We hypothesized that TEA300, CAFF300, and COMBO would increase RTF and power for bench press and squat compared to PLA, but have no effect on 1RM performance. Secondly, we hypothesized that compared to PLA, TEA300, and COMBO would significantly increase energy, focus, and motivation to exercise while decreasing fatigue and RPE, but would not be different from CAFF300.

## Methods

### Participants

Twelve resistance-trained men, ages 20 to 29 years old, were recruited to participate in this study. To be included in the study, participants were required to lift 1.25 times their body weight in the bench press and squat exercises, have regularly trained with loads > 80% of their 1RM for the bench press and squat exercises, and have been currently following a structured resistance exercise program for at least 1 year. Participants were required to have consumed caffeine regularly, with intakes between 100 mg to 300 mg on most days of the week. Participants were excluded if they had a current or recent skeletal muscle injury, were currently taking or had a history of anabolic steroid use, and/or had a diagnosed and untreated metabolic disorder. Participants were asked to abstain from vigorous activity and caffeine 48 h and 24 h prior to testing, respectively. Participants were asked to discontinue use of any dietary supplements (creatine, pre-workouts, etc.) throughout the duration of this study. A washout period of 4 weeks, dependent on the supplements known half-life, was required prior to the start of the study. Participants provided their informed consent after researchers explained the study procedures and potential risks of injury. This study was approved by the Florida State University Institutional Review Board, HSC Number: 2017.21986.

### Procedures

This study utilized a randomized, double-blind, cross-over design and consisted of eight separate laboratory visits. Visit 1 was designed to obtain oral and written informed consent, administer medical history questionnaires, and set up a virtual three-day food and activity log (MyFitnessPal). At the end of visit 1, a standardized meal bar (Dymatize®; 270 kcal, 28 g protein, 24 g carbohydrate, 7 g fat) was provided. Participants were asked to consume the meal bar 60 min prior to arriving for visit 2 following an overnight fast.

### Familiarization trials

During the familiarization visits (visits 2, 3, and 4; ≥ 48 h between visits), participants arrived at the laboratory at 0600–0800 h (60 min after consumption of the standardized meal bar). Upon arrival, participants returned their meal bar wrapper to ensure compliance. Next, height (visit 2) (obtained only at first familiarization trial) and body mass (visits 2–4) were recorded using a digital scale (SECA, California, USA) and wall mounted stadiometer (Detecto, Missouri, USA), respectively. Participants then had their self-reported perceptions measured using 100-mm anchored visual analogue scales (VAS) for energy, focus, motivation to exercise, and fatigue. Participants sat quietly for 90 min, then completed all VAS measures again. Prior to exercise, participants performed a 10-min warmup that included 5 min of walking or jogging on a treadmill (Woodway, Wisconsin, USA) at a self-selected speed and 5 min for any self-selected warm-up they desired. First, bench press and squat (TDS Power Rack) 1RM was determined according to National Strength and Conditioning Association Guidelines [31]. Participants then performed repetitions to failure (RTF) at 70% of their 1RM in both bench press and squat. Participants rested in a seat for 5 min between all 1RM attempts and the transition time before attempting RTF. A TENDO Power Analyzer (TENDO Sports Machines, Slovak Republic) was used to measure power during the RTF in the bench press and squat. RPE was recorded using a modified OMNI-RES 1–10 scale [32] after the completion of the 1RM and RTF for both exercises. Following 10 min of rest after the squat RTF, participants performed a 2-km rowing time trial (Concept2® Model D, Concept2, Inc., Morrisville, VT) at a resistance level of 3–5 in accordance to previously published work [33]. All exercises were supervised by a certified strength and conditioning specialist. Prior to leaving the laboratory, participants were provided another meal bar and instructed to consume it 60 min prior to arrival for the next laboratory visit.

### Experimental trials

For the experimental trials (visits 5–8; 5–8 days between visits), all procedures were identical to the familiarization trials (visits 2–4). Participants consumed each of the following treatments in a random order: (1) Caffeine 300 mg (CAFF300; Compound Solutions Inc., Carlsbad, CA, USA); (2) TeaCrine® 300 mg (TEA300; Compound Solutions Inc., Carlsbad, CA, USA); (3) TeaCrine® + Caffeine (COMBO; 150 mg/150 mg); (4) Placebo 300 mg (PLA, microcrystalline cellulose) prior to the 90-min rest period to allow for peak plasma concentrations to be reached, based on previous research [7]. An investigator administered each treatment in capsule form which was consumed with 12 oz of water. Participants recorded their dietary intake for 3 days each week over the course of the study via MyFitnessPal and were instructed by the researchers to replicate their 24-h diet from the first experimental trial for each subsequent experimental trial. Visits 6, 7, and 8 were identical with the exception of a different supplement being provided prior to the 90-min wait period.

### Exercise protocol

All exercises were performed using a 20-kg Olympic bar with proper technique in accordance to Baechle et al. [31]. A trained researcher/spotter was present for all resistance exercise sessions to ensure proper form and full range of motion. Any repetition that deviated from proper technique was not counted as a successful repetition. Relative time on the rowing ergometer was calculated from a weight-adjustment factor provided by the manufacturer (Concept2, Inc., Morrisville, VT):

$$ {\left[\mathrm{body}\ \mathrm{mass}\ \mathrm{in}\ \mathrm{pounds}/270\right]}^{0.222}\times \mathrm{raw}\ \mathrm{time}\ \left(\mathrm{s}\right) $$

### Statistical analysis

A sample size of 12 was determined through an a-priori power analysis (G*power version 3.1). Sample size was estimated for a one-way comparison of means (matched pairs *t* test) based on a desired statistical power (1 – β) of 0.8 at an α level of 0.05. The effect size used in the calculation (*f* = 0.3) was based on changes in muscular power following caffeine supplementation in a prior study [30].

One-way analysis of variance (ANOVA) was used to examine mean differences in performance measures (muscular strength, endurance, and power) and perceptual data (VAS and RPE) between each of the four treatments. Post hoc analyses were performed with Bonferroni correction to locate differences between means if significance was found. Data are reported as mean ± SD and mean/percent change (95% CI) where appropriate. Significance was accepted as *p* < 0.05 and effect sizes are presented as partial eta squared (η_p_^2^; 0.02 – small effect, 0.13 – medium effect, and 0.26 – large effect). Data were analyzed using SPSS version 25.

## Results

### Descriptive characteristics

Descriptive characteristics of the participants at baseline are provided in Table 1. Fifteen participants were recruited to participate in the study, however, three were unable to comply due to inability to meet inclusion criteria following the start of the intervention.

**Table 1:** Descriptive variables and caffeine intake

Variable	Mean	Range
Age (years)	23.2 ± 3.1	20–29
Height (cm)	177 ± 6	163–185
Body Mass (kg)	83 ± 7	67–92
Relative Bench Press Strength (1-RM/body mass)	1.4 ± 0.2	1.3–1.7
Relative Squat Strength (1-RM/body mass)	1.7 ± 0.2	1.4–2.2
Habitual Caffeine Intake (mg/day)	215 ± 72.7	100–300
Relative Caffeine Intake for Experiments (mg/kg)	3.6 ± 0.3	3.3–4.5

Data presented as mean ± SD

*cm* centimeters, *kg* kilograms, *1-RM/body mass* one-repetition maximum in kg / body mass kg, *mg* milligrams

### Maximal strength (1RM) performance

**Fig. 1:**
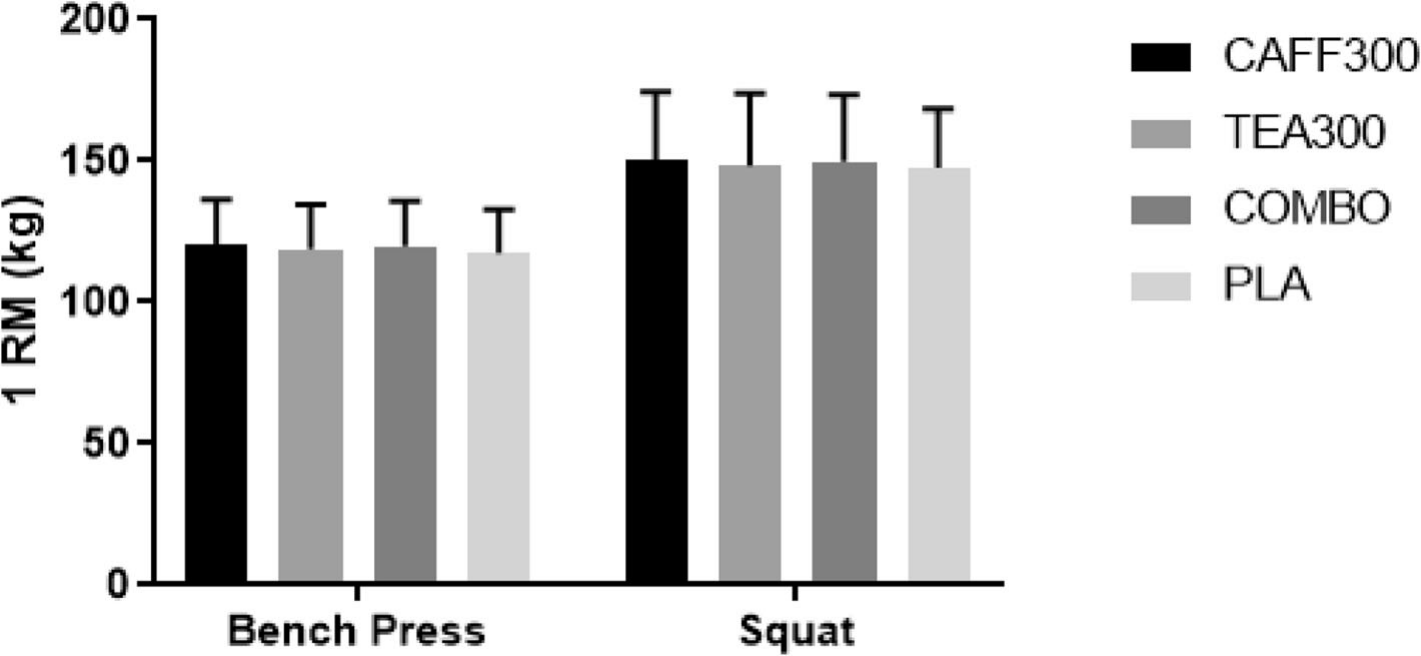
1RM Performance in Bench Press and Squat. kg, kilograms 1 RM; one-repetition maximum; CAFF300, 300 mg of caffeine; TEA300, 300 mg of TeaCrine®; COMBO, 150 mg of caffeine and 150 mg of TeaCrine®; PLA, 300 mg of placebo. Data presented as mean ± SD

Bench press 1RM was 120.0 ± 16.0, 119.0 ± 16.0, 120.0 ± 16.0, and 117.0 ± 16.0 kg for CAFF300, TEA300, COMBO, and PLA, respectively (Fig. 1). The one-way ANOVA revealed significant differences for 1RM bench press performance across groups (F(3,33) = 2.96, *p* = 0.046, η_p_^2^ = 0.21), but subsequent post-hoc analyses revealed no significant group effects (*p* > 0.05) (Table 2).

**Table 2:** Resistance and rowing performance in each treatment condition

	CAFF300	TEA300	COMBO	PLA	*p*
Bench Press 1RM (kg)	120.0 ± 16.0	119.0 ± 16.0	120.0 ± 16.0	117.0 ± 16.0	< 0.05
Bench Press RTF (# reps)	12.0 ± 3.0	12.0 ± 3.0	13.0 ± 3.0	12.0 ± 3.0	0.72
Squat 1RM (kg)	151.0 ± 24.0	149.0 ± 25.0	150.0. ± 24.0	148.0 ± 21.0	0.18
Squat RTF (# reps)	13.0 ± 3.0	11.0 ± 3.0	12.0. ± 4.0	11.0 ± 4.0	0.16
2 k Row TT (s)	478.0 ± 35.1	479.0 ± 39.6	478.6 ± 42.5	483.2 ± 45.6	0.87

Significance (*p* < 0.05) was found in bench press 1RM, but further post-hoc analyses revealed no significant group effects (*p* ≥ 0.05). Data presented as mean ± SD

*CAFF300* 300 mg of caffeine, *TEA300* 300 mg of TeaCrine®, *COMBO* 150 mg of caffeine and 150 mg of TeaCrine®, *PLA* placebo, *1 RM* one-repetition maximum, *Reps* repetitions, *kg* kilograms, *s* seconds, *RTF* reps to failure, *2 k* 2 km, *TT* time-trial

Squat 1RM was 151.0 ± 24.0, 149.0 ± 25.0, 150.0. ± 24.0, and 148.0 ± 21.0 kg for CAFF300, TEA300, COMBO, and PLA, respectively (Fig. 1). There were no significant differences in Squat 1RM across groups (F(1.88,20.71) = 1.88, *p* = 0.18, η_p_^2^ = 0.15) (Table 2).

### Repetitions to failure (RTF) performance

**Fig. 2:**
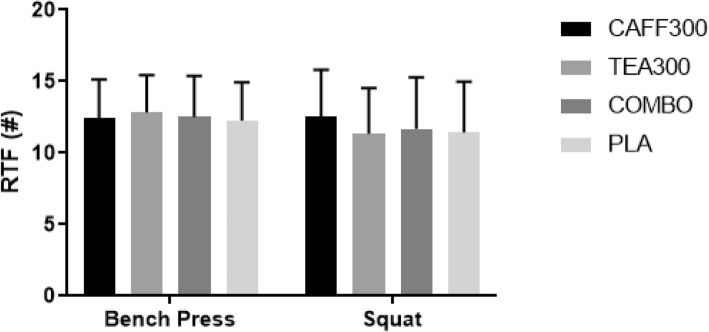
Repetitions to Failure Performance in Bench Press and Squat. RTF, repetitions to failure; #, number of repetitions; CAFF300, 300 mg of caffeine; TEA300, 300 mg of TeaCrine®; COMBO, 150 mg of caffeine and 150 mg of TeaCrine®; PLA, 300 mg of placebo. Data presented as mean ± SD

Bench press RTF were 12.0 ± 3.0, 12.0 ± 3.0, 13.0 ± 3.0, and 12.0 ± 3.0 repetitions for CAFF300, TEA300, COMBO, and PLA, respectively (Fig. 2). There were no significant differences in bench press RTF across groups (F(1.78,19.56) = 0.29, *p* = 0.72, η_p_^2^ = 0.03 (Table 2).

Squat RTF were 13.0 ± 3.0, 11.0 ± 3.0, 12.0. ± 4.0, and 11.0 ± 4.0 repetitions for CAFF300, TEA300, COMBO, and PLA, respectively (Fig. 2). There were no significant differences in squat RTF across groups (F(3,33) = 1.84, *p* = 0.16, η_p_^2^ = 0.14) (Table 2).

### Power/velocity performance

Power and velocity performance data for each treatment group are presented in Table 3. There were no significant differences in peak/average power or peak/average velocity of 1RM or RTF for either exercise (*p* > 0.05).

**Table 3:** Power and velocity for 1 RM and RTF bench press and squat for each treatment

	CAFF300	TEA300	COMBO	PLA	*p*
Peak Power Bench Press 1 RM (W)	355 ± 97	340 ± 117	312 ± 118	301 ± 87	0.35
Peak Velocity Bench 1 RM (m/s)	0.30 ± 0.10	0.29 ± 0.10	0.27 ± 0.10	0.27 ± 0.10	0.45
Average Power Bench RTF (W)	304 ± 47	302 ± 55	292 ± 51	288 ± 50	0.05
Average Velocity Bench RTF (m/s)	0.37 ± 0.03	0.37 ± 0.04	0.35 ± 0.04	0.36 ± 0.04	0.18
Peak Power Squat 1 RM (W)	1092 ± 272	965 ± 333	1010 ± 374	960 ± 198	0.18
Peak Velocity Squat 1 RM (m/s)	0.76 ± 0.20	0.67 ± 0.21	0.69 ± 0.25	0.67 ± 0.14	0.14
Average Power Squat RTF (W)	458 ± 92	440 ± 79	444 ± 79	435 ± 75	0.19
Average Velocity Squat RTF (m/s)	0.44 ± 0.05	0.43 ± 0.04	0.43 ± 0.05	0.43 ± 0.04	0.48

Data presented as mean ± SD. No significant differences were measured (*p* ≥ 0.05)

*CAFF300* 300 mg of caffeine, *TEA300* 300 mg of TeaCrine®, *COMBO* 150 mg of caffeine and 150 mg of TeaCrine®, *PLA* placebo, *1 RM* one-repetition maximum, *Reps* repetitions, *RTF* reps to failure, *W* watts, *m/s* meters per second

### 2-km row performance

Relative row times for the 2 km, time-trial were 478.0 ± 35.1, 479.0 ± 39.6, 478.6 ± 42.5, and 483.2 ± 45.6 s for CAFF300, TEA300, COMBO, and PLA, respectively. There were no significant differences in relative row time across groups (F(3,33) = 0.24, *p* = 0.87, η_p_^2^ = 0.02) (Table 2).

### Perceptual response

**Fig. 3:**
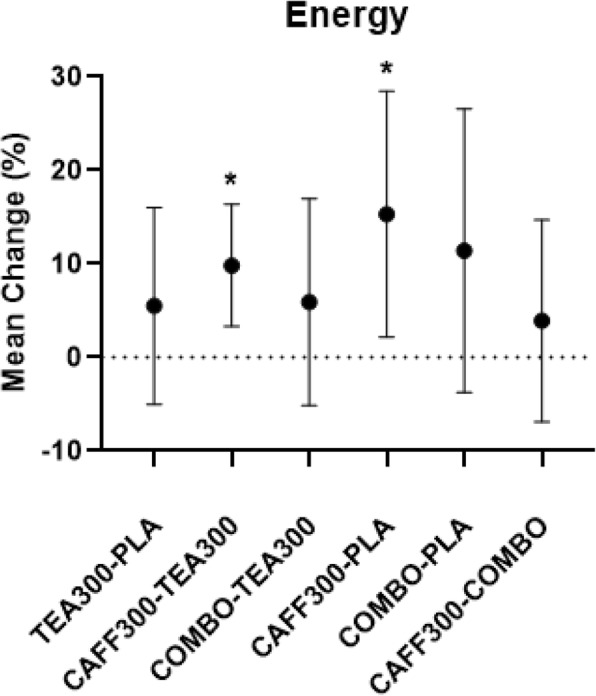
Perception of Energy. Differences between TEA300, CAFF300, COMBO, and PLA from Baseline to 90 min Post-Treatment in Perception of Energy CAFF300, 300 mg of caffeine; TEA300, 300 mg of TeaCrine®; COMBO, 150 mg of caffeine and 150 mg of TeaCrine®; PLA, 300 mg of placebo. TEA300-PLA, TEA300 vs. PLA; CAFF300-TEA300, CAFF300 vs. TEA300; COMBO-TEA300, COMBO vs. TEA300; CAFF300-PLA, CAFF300 vs. PLA; COMBO-PLA, COMBO vs. PLA; CAFF300-COMBO, CAFF300 vs. COMBO; cm, centimeters. *denotes significantly different (*p* < 0.05). Data presented as mean/percent change (95% CI)

**Fig. 4:**
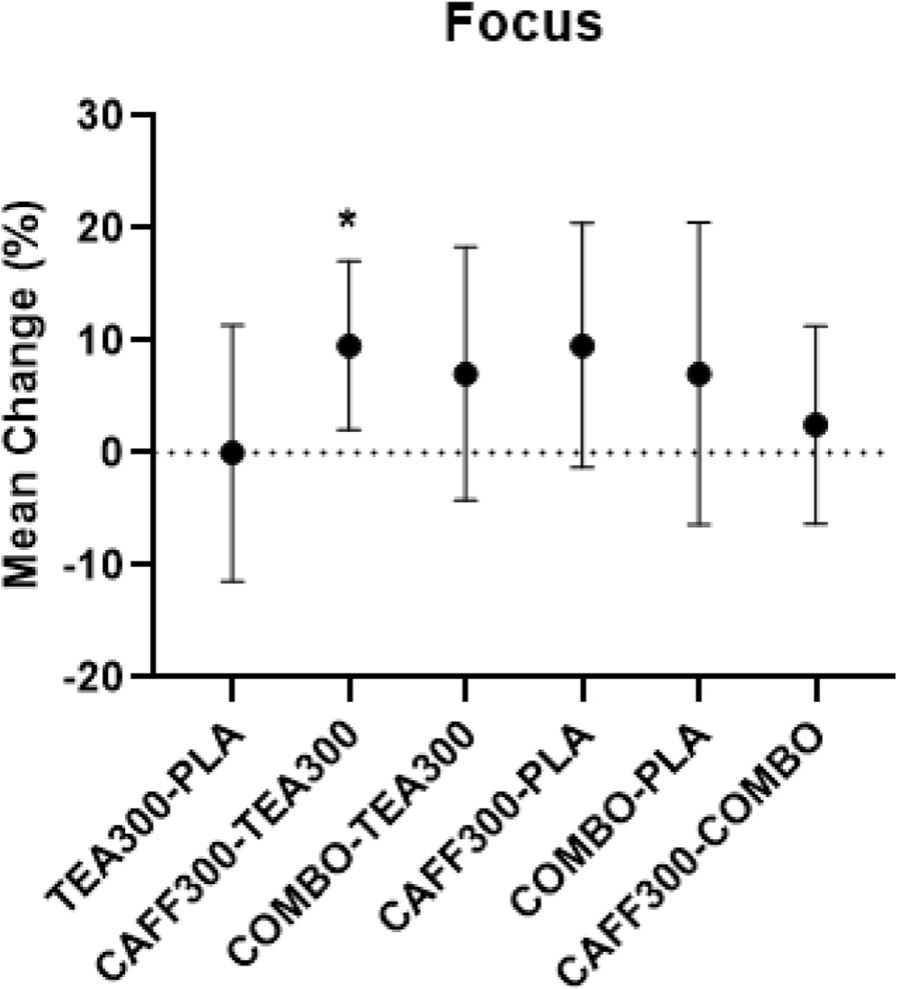
Perception of Focus. Mean Differences between TEA300, CAFF300, COMBO, and PLA from Baseline to 90 min Post-Treatment in Perception of FocusCAFF300, 300 mg of caffeine; TEA300, 300 mg of TeaCrine®; COMBO, 150 mg of caffeine and 150 mg of TeaCrine®; PLA, 300 mg of placebo. TEA300-PLA, TEA300 vs. PLA; CAFF300-TEA300, CAFF300 vs. TEA300; COMBO-TEA300, COMBO vs. TEA300; CAFF300-PLA, CAFF300 vs. PLA; COMBO-PLA, COMBO vs. PLA; CAFF300-COMBO, CAFF300 vs. COMBO; cm, centimeters. *denotes significantly different (*p* < 0.05). Data presented as mean/percent change (95% CI)

**Fig. 5:**
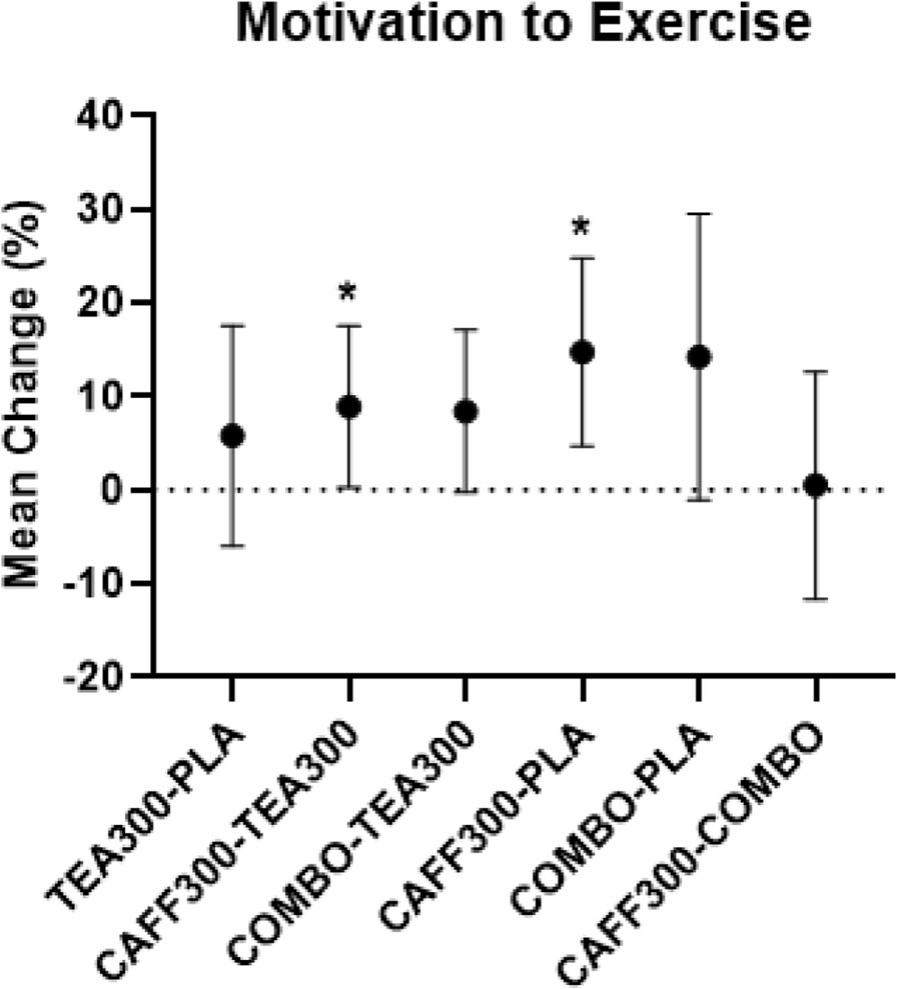
Perception of Motivation. Mean Differences between TEA300, CAFF300, COMBO, and PLA from Baseline to 90 min Post-Treatment in Perception of Motivation.CAFF300, 300 mg of caffeine; TEA300, 300 mg of TeaCrine®; COMBO, 150 mg of caffeine and 150 mg of TeaCrine®; PLA, 300 mg of placebo. TEA300-PLA, TEA300 vs. PLA; CAFF300-TEA300, CAFF300 vs. TEA300; COMBO-TEA300, COMBO vs. TEA300; CAFF300-PLA, CAFF300 vs. PLA; COMBO-PLA, COMBO vs. PLA; CAFF300-COMBO, CAFF300 vs. COMBO; cm, centimeters. *denotes significantly different (*p* < 0.05). Data presented as mean/percent change (95% CI)

**Fig. 6:**
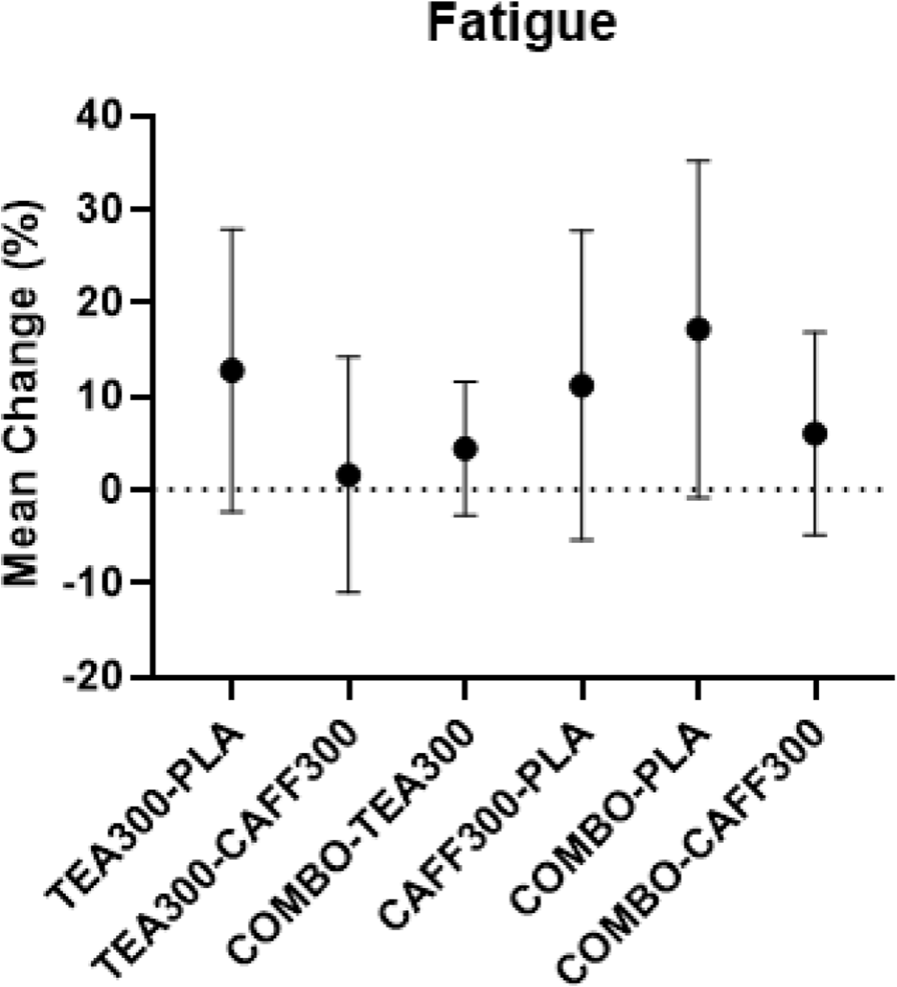
Perception of Fatigue. Mean Differences between TEA300, CAFF300, COMBO, and PLA from Baseline to 90 min Post-Treatment in Perception of Fatigue.CAFF300, 300 mg of caffeine; TEA300, 300 mg of TeaCrine®; COMBO, 150 mg of caffeine and 150 mg of TeaCrine®; PLA, 300 mg of placebo. TEA300-PLA, TEA300 vs. PLA; TEA300-CAFF300, TEA300 vs. CAFF300; COMBO-TEA300, COMBO vs. TEA300; CAFF300-PLA, CAFF300 vs. PLA; COMBO-PLA, COMBO vs. PLA; COMBO-CAFF300, COMBO vs. CAFF300; cm, centimeters. Data presented as mean/percent change (95% CI)

**Fig. 7:**
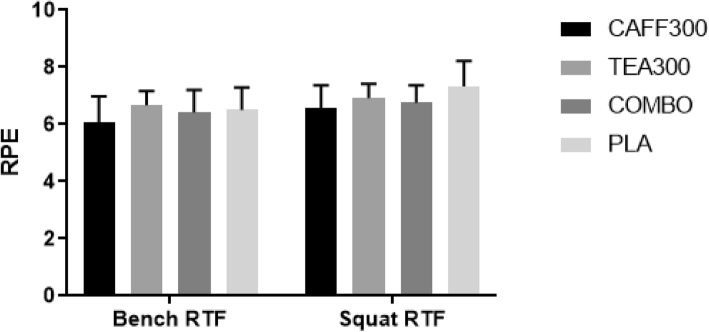
Rating of Perceived Exertion After Bench Press and Squat. RPE, rating of perceived exertion; RTF, repetitions to failure; CAFF300, 300 mg of caffeine; TEA300, 300 mg of TeaCrine®; COMBO, 150 mg of caffeine and 150 mg of TeaCrine®; PLA, 300 mg of placebo. Data presented as mean ± SD

There was a significant group effect for energy, focus, motivation to exercise, and fatigue from baseline to 90 min post-treatment. Post-hoc analyses revealed mean differences in energy between baseline and 90 min post-treatment were significantly higher in CAFF300 compared to TEA300 and PLA (+ 9.8%, 95% CI [3.3–16.4%], *p* < 0.01; + 15.3%, 95% CI [2.2–28.5%], *p* < 0.02, respectively) (Fig. 3). Similarly, mean differences in focus between baseline and 90 min post-treatment were significantly higher in CAFF300 compared to TEA300 (+ 9.6%, 95% CI [2.1–17.1%], *p* = 0.01) (Fig. 4). Additionally, mean differences in motivation to exercise between baseline and 90 min post-treatment were significantly higher in CAFF300 compared to TEA300 (+ 8.9%, 95% CI [0.2–17.6%], *p* = 0.04) and PLA (+ 14.8%, 95% CI [4.7–24.8%], *p* < 0.01) (Fig. 5). There were no significant mean differences from baseline to 90 min post-treatment for fatigue levels (*p* > 0.05) (Fig. 6). There was a significant group effect in squat RTF RPE, but not bench press RTF RPE. However, post-hoc analyses revealed no significant differences between groups. Interestingly, RPE was trending towards significance in CAFF300 and COMBO compared to PLA (− 7.5, *p* = 0.07, η_p_^2^ = 0.18; − 5.8%, *p* = 0.07, η_p_^2^ = 0.14, respectively) during Squat RTF (Fig. 7).

### Nutritional intake

There were no significant differences (F(3,33) = 1.69, *p* = 0.19, η_p_^2^ = .13) in total caloric intake 24 h before experimental trials between groups (CAFF300: 2112 ± 552, TEA300: 2391 ± 495, COMBO: 2286 ± 438, PLA: 2346 ± 594 kcals).

## Discussion

The present study is the first to examine the efficacy of TeaCrine®, a caffeine-like compound, on maximal muscular strength, endurance, and power performance in resistance trained-men. The primary findings were: 1) CAFF300, TEA300 and COMBO had no significant effect on bench press 1RM, squat 1RM, bench press RTF, squat RTF, and power compared to PLA; 2) CAFF300 had significant increases in self-reported energy, focus, and motivation to exercise, but not RPE, compared to TEA300, COMBO, and PLA. We accept our hypothesis that there would be no differences in bench press and squat performance between groups, but reject our hypothesis that CAFF300, TEA300, and COMBO would increase RTF and power performance compared to PLA. Additionally, we reject that compared to PLA, TEA300 and COMBO would significantly increase energy, focus, and motivation to exercise while decreasing fatigue and RPE, but would not be different from CAFF300.

### Strength

Bench press 1RM was 2.2% higher in the CAFF300 group compared to PLA (*p* = 0.19, η_p_^2^ = 0.007) and, while not significant, was descriptively similar to previous research that reported significant increases in performance with caffeine ingestion [22, 26]. Beck et al. [26] reported a 2.1% (+ 2.1 kg) increase in bench press 1RM following ingestion of a 201 mg caffeine containing supplement (approximately 2.4 mg/kg) compared to placebo. However, there were differences in the sample size (*n* = 37 vs *n* = 12). Additionally, the supplement used contained over 10 ingredients, thus the improvements cannot be solely attributed to caffeine. Similarly, Goldstein et al. [22] reported a significant 1.5% increase in bench press 1RM following ingestion of 6 mg/kg caffeine compared to placebo in resistance trained females, which was similar to the outcome of the current study, but ours did not meet significance. Differences in Goldstein et al. and the current study were possibly due to the differing characterization of training status and dose administration between studies. Goldstein recruited trained females able to lift 70% of their body mass, whereas, we recruited trained males capable of lifting 125% of their body mass, leading to possible discrepancies in training status between studies. It is possible that unknown sex differences may be a driving factor to discrepancy in outcomes, which is difficult to speculate due to a lack of resistance exercise research including female participants. Also, when adjusting caffeine content in relation to body mass, the present study utilized a lower caffeine dose per body mass (~ 3.6 mg/kg compared to 6 mg/kg). As such, our treatment dose may not have met the threshold to see significant improvements in bench press 1RM, however, 3 mg/kg is thought of as the threshold to elicit ergogenic effects in resistance exercise outcomes [15]. A lower dose of caffeine (3.6 ± 0.3 mg/kg) was used in the current study due to a lack of data on the drug-drug interactions between caffeine and TeaCrine®, to examine potential ergogenic effects at a known ergogenic dose while mitigating potential unwanted side effects that have been reported at higher dosages. Additionally, absence of any ergogenic effect may have been a result of the administration of a meal bar preceding treatment ingestion, which is in contrast with the previously mentioned studies in which all consumed the caffeine related supplements in a fasted state. Prior research has reported the absorption of caffeine is slowed following ingestion of food in conjunction with reduced peak plasma concentrations, resulting in a possible treatment concentration below the ergogenic threshold, that otherwise would have been reached had the treatment been given in a fasted state [34]. However, this protocol was chosen specifically to be more applicable to those that do not fast prior to intense workouts. Clearly, more research is warranted to examine the effects of dosing strategies of TeaCrine®, caffeine, and a combination of these ingredients in addition to fasted vs. fed states on bench press 1RM.

Similarly, for squat 1RM, performance in CAFF300 was 1.8% and 1.6% higher compared to PLA and TEA300, respectively. Both performance effects were nonsignificant which is in agreement with previous literature [23, 25, 26].

### Endurance

In the present study, no significant change was reported for RTF at 70% 1RM from any of the four treatments. However, other studies have reported a significant effect for RTF with caffeine administration [10, 16, 17]. Duncan et al. [17] reported that 5 mg/kg caffeine ingested in the fasted state 60 min pre-exercise increased RTF at 60% 1RM in the bench press and back squat compared to placebo in resistance trained men (*n* = 9) and women (*n* = 2). Potential discrepancies with the present study are likely due to the amount of caffeine administered and the treatments consumed in a non-fasted state. Duncan et al. gave a dose of 5 mg/kg caffeine compared to the mean dose of ~ 3.6 mg/kg in the present study. Current literature supports a potential ergogenic effect of caffeine with a dose of 3–9 mg/kg [15, 35]; as such, the dose administered in the present study may be on the lower threshold for ergogenic effect. Additionally, the participants may have been more trained than in the current study, as Duncan et al. utilized resistance trained individuals (9 ± 5.5 yrs. experience) with competency in Olympic lifting techniques and programing > 10 h per week of strength and conditioning activities.

Astorino et al. [10] reported that RTF in the leg press exercise at 80% 1RM increased after ingesting 6 mg/kg caffeine 60 min before exercise compared to placebo in resistance trained men (Caffeine: 15.71 ± 6.88 repetitions vs PLA: 14.07 ± 6.17 repetitions, *p* < 0.05). Interestingly, they also reported no differences in the bench press RTF between caffeine and a placebo which is in agreement with the present study. Researchers were unable to determine the mechanism that elicited increased leg press performance with caffeine consumption with no ergogenic effects in the other exercises. Astorino et al. hypothesized that possible increases may have been due to decrements in performance in the placebo trial due to caffeine withdrawal. Six of the nine (66.67%) participants who revealed performance increases during the caffeine trial were described as heavy caffeine users (daily intake > 225 mg/day). During their placebo trial, in which no caffeine was consumed, those participants exhibited caffeine withdrawal symptoms (headaches and lethargy) which led to reductions in performance. While caffeine has been shown to have both an effect [10, 11, 16–19, 22, 26] and no effect [22, 23, 25, 26, 36, 37] on resistance exercise performance, the present study revealed no effect of caffeine, TeaCrine®, or a combined dose of caffeine and TeaCrine® on bench press or squat RTF performance.

### Power

Peak and mean power and velocity were not significantly different between any of the treatment groups. These findings are in contrast with previous literature, which has reported increases in peak power and mean bar velocity in the bench press and squat exercises with caffeine supplementation [28–30]. Mora-Rodriguez et al. [30] examined the effects of 3 mg/kg of caffeine ingested 60 min pre-exercise in 12 resistance trained men. The authors reported significant increases in mean bar velocity at a load of 75% 1RM during the bench press and squat in caffeine compared to placebo. In a follow-up intervention, the authors administered 6 mg/kg of caffeine in 13 resistance trained men and reported significant increases in mean bar velocity during the squat at loads of 25%, 50%, and 75% 1RM in the caffeine group (5.4–8.5%, *p* = 0.037–0.001) compared to placebo [29]. Mora-Rodriguez et al. utilized a dose of 6 mg/kg compared to the ~ 3.6 mg/kg in the present study. Similarly, Pallarés et al. [28] reported doses of 6 and 9 mg/kg were effective at increasing mean velocity in the bench press and peak power in the squat. Differences in outcomes may again be a result of an insufficient dose of caffeine and/or TeaCrine® and treatments ingested in a non-fasted state. While 3 mg/kg caffeine resulted in significant increases in mean bar velocity in the bench press and squat [30], the present study’s ~ 3.6 mg/kg caffeine did not. This may be a result of differences in training status. Goldstein et al. [38] remarks that caffeine does not appear to be effective for non-trained individuals due to variability of performance typical of untrained individuals. The present study categorized training status as an ability to lift 125% of their body weight in both exercises and to have been following a high-intensity training program for > 1 year. Conversely, Mora-Rodriguez et al. [29, 30] and Pallarés et al. [28] recruited a more trained population of highly resistance trained men with a training experience of 7.1 ± 3.5 yrs., 7.2 ± 2.4 yrs., and 7.1 ± 3.5 yrs., respectively. Similarly, the studies eliciting an ergogenic effect of caffeine reported by Astorino et al. [35] consisted primarily of trained athletes, including competitive cyclists, football players, competitive swimmers, and “elite” athletes, not young, trained men which was the population examined in the present study.

### Rowing

There were no significant differences in rowing time trial performance across treatments, disagreeing with previous literature, which has reported increased performance from caffeine consumption [39, 40]. Bruce et al. [39] administered 6 and 9 mg/kg caffeine in well-trained male rowers 45 min before a 2-k rowing time trial (TT) and reported an increase in TT completion by 1.3% in the 6 mg/kg group, but no differences in the 9 mg/kg group compared to placebo. Anderson et al. [40] administered the same doses of caffeine in competitive oarswomen 60 min pre 2 k TT and reported increases in TT completion by 1.3% with 9 mg/kg, but not with 6 mg/kg compared to placebo. Difference in outcomes to the present study are likely attributed to differences in training status. Both Anderson et al. and Bruce et al. employed competitive rowers, whereas the present study had no set training parameters for rowing, resulting in possible high variability in performances (mean CV = 8.3%). Additionally, caffeine in the previous studies was consumed 45–60 min prior to exercise and was the main performance measure. However, in the present study, participants consumed their treatment ~ 150 min prior to performing the rowing TT and was the last exercise to be performed. As such, it is possible that any potential ergogenic effect of caffeine was diminished due to the length of time preceding the TT, or to fatigue from the previous exercises.

### Cognitive perceptions

Measurements of cognitive perceptions via VAS revealed significant increases in mean differences for energy, focus, and motivation to exercise from baseline to 90 min post-treatment in CAFF300 compared to PLA and TEA300, but not COMBO. While neither TEA300 or COMBO treatment resulted in significant differences to PLA, there were trends for mean differences from baseline to 90-min post-treatment in measures of motivation (COMBO > TEA300; *p* = 0.06, η_p_^2^ = 0.21) and fatigue (COMBO < PLA; *p* = 0.07, η_p_^2^ = 0.34) which is similar to previous research that reported no significant effects or trends towards significance with TeaCrine® consumption [4, 5]. In contrast, Ziegenfuss et al. [6] utilized a two-part approach of TeaCrine® supplementation on subjective cognitive parameters. In the first part, energy, focus, and motivation to exercise significantly increased from baseline with no dose-response effect in TeaCrine® (200 mg vs 400 mg) compared to placebo, with significant group x time effects for energy (TeaCrine®: + 8.6% vs PLA: − 5.7%, *p* = 0.049) and fatigue (TeaCrine®: − 6.7% vs PLA: − 1.3%, *p* = 0.05). In the second part, significant increases in concentration were reported in TeaCrine® (200 mg) compared to placebo (TeaCrine®: + 2.4% vs PLA: − 1.3%, *p* = 0.07). With limited data available, more research is needed to understand the possible effects of TeaCrine® on various subjective measures of cognitive perception. While there were no significant effects across treatments on RPE during RTF, there were trends for a reduction in RPE for squat RTF with CAFF300 compared to PLA (*p* = 0.07, η_p_^2^ = 0.18) and COMBO to PLA (*p* = 0.07, η_p_^2^ = 0.14). A meta-analysis by Doherty et al. [41] reported that caffeine at doses of 4–10 mg/kg ingested 30–150 min before constant rate exercise (cycling, running, swimming; 50–125% VO_2max_) reduced RPE by 5.6 ± 5.3%, with RPE accounting for 29% of variance in performance improvement during exercise.

Muscle pain is thought to influence RPE during exercise. One of the main metabolites thought to cause pain during exercise is adenosine, which can be antagonized through caffeine ingestion. This antagonism is believed to be responsible for the analgesic properties of caffeine during whole-body exercise, which is speculated to decrease an individual’s RPE [42–44]. While these findings do not include resistance exercise, they can be broadly applied to interpret a potential effect of caffeine on RPE in resistance exercise. Our findings differ from Duncan et al. [17] in which the authors reported a reduction in RPE compared to PLA (Mean difference: − 8.4%, *p* = 0.03) for RTF in the bench press exercise at 60% 1RM. Conversely, Green et al. [18] reported increased RTF in the squat at a load equal to the maximum reps performed for 10 reps, but no differences in RPE with caffeine supplementation compared to placebo. The present study utilized a modified RPE scale for resistance exercise to more accurately identify RPE at the active muscle [32]. The results of the present study may differ from the results of the previous research due to a difficulty of accurately measuring exertion during short-term high-intensity exercise [35, 45]. Differences in RPE scales make it difficult to compare the results of the current study to previous literature. Duncan et al. [17] utilized a traditional Borg scale, whereas Green et al. [18] used the same scale as the present study. Therefore, future investigations must consider the challenges in accurately measuring RPE and comparing perceived exertion using different scales.

### Limitations

While measures were taken to ensure strict control over the study, there were some limitations. First, to our knowledge, this is the first study to examine the effects of TeaCrine® on resistance exercise performance. As such, we are unable to compare the results of the TeaCrine® containing treatments, TEA300 and COMBO, on resistance exercise performance to other studies which used TeaCrine®. The amount of caffeine used in this study, 300 mg (3.6 ± 0.3 mg/kg) is within the noted ergogenic range (3–9 mg/kg) [15, 35], but may be at the lower end, as such, a higher dose of 5–6 mg/kg may be needed in order to elicit ergogenic effects in this population following ingestion of a meal bar. The potential ergogenic effects of a higher dose in combination with a higher dose of TeaCrine®, or a higher dose of TeaCrine® only, may also be needed. For RTF, participants were instructed to perform the exercise until they reached volitional failure and could not perform another repetition. However, it is possible that volitional failure preceded true muscular failure. Additionally, consumption of a meal bar preceding treatment ingestion may have altered the pharmacokinetics of each treatment, thus requiring higher doses of supplementation, than has been previously reported, to show ergogenic benefit. It is important to note that the same bar was consumed prior to all trials, and thus the influence of the bar would be the same across all conditions. We felt this study design provided novel and applicable information for general population and coaches, as consuming food prior to exercise has known benefits [46]. Therefore, determining the dosage that is required in conjunction with food and utilizing a fasted state model are important considerations for future research. Additionally, a matched dose of caffeine was implemented as a quasi-control, as many studies involving multi-ingredient performance supplements with caffeine blends do not do this.

## Conclusion

In conclusion, CAFF300, TEA300 and COMBO (when consumed 90 min pre-exercise) had no significant effect on 1RM, RTF, power, or velocity in the bench press or squat in resistance-trained men. CAFF300 improved measures of focus, energy, and motivation to exercise but TEA300, COMBO, and PLA did not. Future studies should utilize a higher dose of TeaCrine® by itself as well as in combination with caffeine.

## Data Availability

The datasets used and/or analyzed during the current study are available from the corresponding author upon reasonable request.

## Abbreviations

1RM: 1 repetition maximum
CAFF300: 300 mg of caffeine
COMBO: 150 mg of caffeine + 150 mg of TeaCrine®
PLA: Placebo
Rep: Repetition
RPE: Rating of perceived exertion
RTF: Repetitions to failure
TEA300: 300 mg of TeaCrine®
VAS: Visual analog scale
